# Molecular Detection and Phylogenetic Characterization of CPV-2c in Apparently Healthy Domestic Cats in the Guadalajara Metropolitan Area, Mexico

**DOI:** 10.3390/vetsci13090977

**Published:** 2026-09-17

**Authors:** Sandra Del Carmen Robles Gil-Bernal, Darwin Elizondo-Quiroga, Jeannette Barba-León, Claudia Lisette Charles-Niño, Ana Samatzin Leal-Pimentel, Jorge Gaona Bernal, César Pedroza-Roldán

**Affiliations:** 1 Centro Universitario de Ciencias Biológicas y Agropecuarias, Departamento de Medicina Veterinaria, Universidad de Guadalajara, Zapopan 45200, Jalisco, Mexico; sandra.roblesgil@academicos.udg.mx (S.D.C.R.G.-B.); ana.leal4371@alumnos.udg.mx (A.S.L.-P.); 2 Centro Universitario de Ciencias Biológicas y Agropecuarias, Doctorado en Ecofisiología y Recursos Genéticos, Universidad de Guadalajara, Zapopan 45200, Jalisco, Mexico; 3 Unidad de Biotecnología Médica y Farmacéutica, Centro de Investigación y Asistencia en Tecnología y Diseño del Estado de Jalisco, Asociacion Civil, Guadalajara 44270, Jalisco, Mexico; delizondo@ciatej.mx; 4 Departamento de Salud Pública, Centro Universitario de Ciencias Biológicas y Agropecuarias, Universidad de Guadalajara, Zapopan 45200, Jalisco, Mexico; jeannette.barba@academicos.udg.mx; 5 Department of Oral Biology, College of Dentistry, University of Florida, Gainesville, FL 32610, USA; c.charlesnino@ufl.edu; 6 Departamento de Microbiología y Patología, Centro Universitario de Ciencias de la Salud, Universidad de Guadalajara, Guadalajara 44340, Jalisco, Mexico; jorge.gaona@academicos.udg.mx

**Keywords:** carnivore protoparvovirus 1, molecular epidemiology, risk factors, disease reservoirs, host specificity

## Abstract

Carnivore protoparvovirus 1 variants (CPV-2a, CPV-2b, CPV-2c) have progressively expanded their host range to domestic cats (Felis catus), causing subclinical infection or panleukopenia-like disease. CPV-2c dominates among dogs in Western Mexico, but its presence in the local feline population had not been investigated. A cross-sectional survey was conducted (2018–2022) in 210 apparently healthy domestic cats from five municipalities of the Guadalajara Metropolitan Area, Jalisco, Mexico. Genomic DNA from EDTA-anticoagulated blood was screened by generic protoparvovirus PCR; positive samples were characterized by CPV-2-specific VP2 PCR and Sanger sequencing, with variant classification based on VP2 residue 426, and phylogenetic relationships assessed by maximum-likelihood analysis (T92+I model; 1000 bootstrap replicates). Carnivore protoparvovirus 1 DNA was detected in 14/210 cats (6.7%); 13/14 sequenced isolates were classified as CPV-2c (Glu426, >99% nucleotide identity), clustering with regional canine strains and showing greatest affinity with Uruguayan isolates. In a Firth bias-reduced multivariable model adjusting for age and vaccination status, shared litter box use remained independently associated with detection (adjusted OR 18.03; 95% CI 3.30–98.58); the age association was attenuated after adjustment, suggesting incomplete vaccination, rather than age itself, as the more proximal risk factor. Free-roaming/outdoor access and hunting behavior showed direct crude associations with detection (OR 3.82 and 7.22, respectively). These findings show that cats can harbor CPV-2c DNA in blood without clinical signs, phylogenetically linked to local canine strains, underscoring the need for integrated CPV-2 surveillance across companion species; however, whether infected cats can transmit the virus remains undetermined.

## Simple Summary

Cats can be infected by parvoviruses related to those that cause severe intestinal disease in dogs, but most infected cats show no symptoms. This study investigated whether healthy pet cats in the Guadalajara area of Mexico carry this virus, since a related virus is already common among local dogs. Blood samples from 210 apparently healthy cats were tested using molecular laboratory methods that detect viral genetic material. The virus was found in about 7 out of every 100 cats, even though none appeared sick. Genetic analysis showed the virus found in cats was nearly identical to the virus already circulating in local dogs, and closely related to strains previously reported in South America. Cats that shared a litter box with other cats, spent time outdoors, or hunted were more likely to test positive, suggesting that contact with contaminated environments spreads the virus. These findings show that outwardly healthy cats can carry this virus in their blood without any signs of illness, meaning disease-monitoring programs, which currently focus almost entirely on dogs, should also include cats. However, this study could not determine whether infected cats can pass the virus to other animals, which future research should investigate.

## 1. Introduction

Carnivore protoparvovirus 1 (which includes CPV-2 and FPV) comprises small, non-enveloped ssDNA viruses that preferentially replicate in rapidly dividing cells, particularly intestinal crypts, bone marrow, and lymphoid tissues [1,2]. CPV-2 emerged in dogs in the late 1970s from an FPV-like ancestor after a handful of mutations in the VP2 capsid protein changed transferrin receptor binding and expanded host range to canids [1,3,4]. Subsequent evolution generated three antigenic variants distinguished by residue 426 of VP2 CPV-2a (Asn), CPV-2b (Asp), and CPV-2c (Glu) all of which regained the ability to infect cats in addition to dogs [5,6]. CPV-2c, first identified in Italy around 2000, has since become a major globally circulating variant, currently predominating not only in many regions of South America (Uruguay, Argentina, Brazil, and Chile) but also across large parts of Asia and Europe, including Thailand, China, and other countries where it has emerged as the dominant or a rapidly expanding antigenic type [7,8,9,10,11,12,13]. Notably, CPV-2c has shown a particular propensity, relative to CPV-2a and CPV-2b, to infect domestic cats and induce clinical disease resembling feline panleukopenia, as demonstrated experimentally and in natural infections [14]. Phylogenetic analyses indicate that these South American CPV-2c strains cluster in a Europe-derived “Europe I” clade and have diversified locally following intercontinental introductions [10]. In Mexico, multiple molecular surveys have shown that CPV-2c is the dominant, and in some series exclusive, canine variant, with western Mexico and the Guadalajara Metropolitan Area showing sustained CPV-2c circulation and >99% nucleotide identity (based on VP1/VP2 gene and whole-genome sequence comparisons) to European and South American strains [15,16,17,18].

Although FPV remains the main cause of feline panleukopenia, accumulating evidence shows that CPV-2 variants (2a–2c) can infect domestic cats, leading to either classical panleukopenia or subclinical infection [19]. Studies in the UK and Europe have documented fecal shedding of CPV-2a/2b by clinically healthy shelter cats, sometimes in up to one-third of animals, with viral sequences closely related to co-circulating canine strains, raising the possibility that cats could contribute to viral maintenance in multi-species environments; however, definitive reservoir status would require additional evidence of sustained shedding and demonstrated transmission [20,21]. More recently, CPV-2c has been detected in cats in India, Egypt, and other regions, confirming the expanding feline host range of this variant [22,23,24,25].

Risk factors that favor parvovirus transmission in cats include group housing, multi-animal households, shared litter boxes, outdoor access, and young age, all of which increase contact with infectious feces or contaminated fomites [21]. In Mexico, however, virtually all molecular epidemiology data on CPV-2c derive from dogs, and no prior study has assessed CPV-2 or specifically CPV-2c in clinically healthy domestic cats, nor compared feline sequences with local canine strains [15,16,17,18]. This knowledge gap, set against a background of high CPV-2c circulation in dogs and frequent dog/cat cohabitation in the GMA, provides the epidemiological rationale for investigating CPV-2c in the feline population of the region. Therefore, the aims of the present study were to: (i) determine the molecular detection rate of Carnivore protoparvovirus 1 in apparently healthy domestic cats from the Guadalajara Metropolitan Area, Jalisco, Mexico; (ii) characterize the circulating variant(s) based on amino acid residue 426 of the VP2 capsid protein; (iii) establish the phylogenetic relationships of the detected feline sequences to regional canine and international CPV-2c reference sequences; and (iv) identify management and demographic factors associated with CPV-2c detection in the sampled feline population.

## 2. Materials and Methods

### 2.1. Study Design and Sample Size

A cross-sectional molecular survey was conducted between 2018 and 2022 in domestic cats (Felis catus) from the Guadalajara Metropolitan Area (GMA), Jalisco, Western Mexico, comprising the municipalities of Guadalajara, Zapopan, San Pedro Tlaquepaque, Tlajomulco de Zúñiga, and Tonalá. The minimum sample size was estimated in OpenEpi v3.03 for a descriptive proportion study, assuming a finite source population of 100,000 cats (National Survey of Companion Animals, INEGI), an expected prevalence of 10%, an absolute precision (margin of error) of 5%, a 95% confidence interval, and a design effect of 1.0. Because no prior data on CPV-2/CPV-2c prevalence in domestic cats were available for this region, an expected prevalence of 10% was adopted as a conservative planning estimate, informed by the wide range of subclinical CPV/FPV detection rates reported internationally in apparently healthy cats (16.7–48.7%); [20,21,26] and by the CPV-2c circulation already documented in the local canine population [15,16]. The minimum required sample size was 139; after a 10% adjustment for expected sample loss, 153 samples were deemed sufficient. Enrollment followed a convenience multi-site approach, acknowledged as a potential source of selection bias. Of 260 blood samples collected, 50 were excluded prior to molecular analysis (insufficient DNA yield, n = 22; sample degradation from inadequate cold-chain handling, n = 15; failed β-globin PCR, n = 8; incomplete epidemiological record, n = 5). As all exclusion causes were technical or logistical, the potential for exclusion-related selection bias cannot be completely ruled out because excluded samples could not be assessed for CPV-2c status. The remaining 210 samples constituted the final analytical dataset.

### 2.2. Animal Enrollment and Data Collection

Cats were enrolled at the Small Animal Veterinary Hospital of the Universidad de Guadalajara, municipal spay/neuter mobile units (Centro de Salud y Control Animal of Guadalajara and San Pedro Tlaquepaque), a feral cat rescue center, and private households, with no restrictions on age, sex, breed, or vaccination status. At enrollment, a structured questionnaire recorded age, sex, breed, body weight, outdoor access, free-roaming and hunting behavior, litter box use, multi-animal household exposure, vaccination/deworming history, and sterilization status. A general physical examination documented physiological parameters, mucous membrane condition, and peripheral lymph node status.

### 2.3. Blood Collection and DNA Extraction

Whole blood, rather than fecal samples, was selected primarily because it was concurrently collected for a parallel feline toxoplasmosis investigation in the same cohort, and because standardized fecal collection was logistically difficult across the multiple community and shelter settings involved. No prior studies had specifically validated whole blood as a diagnostic matrix for subclinical CPV-2/CPV-2c detection in cats; this methodological choice and its implications, including likely underestimation of intestinal shedding, are further addressed in the Limitations section. One milliliter of whole blood was collected by jugular venipuncture. Aliquots of 200 μL were transferred to sterile Eppendorf microcentrifuge tubes containing approximately 20–30 μL of 0.5 M disodium EDTA solution (pH 8.0), a concentration routinely used as anticoagulant in molecular diagnostic protocols, and transported under refrigeration to the Veterinary Immunology Laboratory, Small Animal Veterinary Hospital, Universidad de Guadalajara. Genomic DNA was extracted using the GF-1 Blood DNA Extraction Kit (Vivantis Technologies, Subang Jaya, Malasya) per manufacturer instructions, evaluated for integrity by 1% agarose gel electrophoresis, and quantified at 260 nm (NanoDrop 2000, Thermo Fisher Scientific, Waltham, MA, USA). Samples were stored at −20 °C until use.

### 2.4. DNA Quality Validation and Molecular Screening

The amplifiability of each DNA sample was confirmed by a β-globin PCR (140-bp fragment) prior to viral testing [27,28]. Validated samples were then screened for Carnivore protoparvovirus 1 (encompassing both FPV and CPV-2) using a generic PCR (primers FTA1/FTA2, designed in-house for this study) targeting a conserved 150-bp region shared by both viral genomes. Primers were designed to target regions of high nucleotide conservation between FPV and CPV-2 VP2 sequences (~96% homology), identified through multiple-sequence alignment of GenBank reference sequences. Primer specificity and the absence of problematic secondary structures (self-dimers, hairpins) were verified in silico using NCBI Primer-BLAST, 2.5.0 virtual PCR prior to laboratory testing. The assay was subsequently optimized empirically by testing an annealing temperature gradient (±5 °C around the calculated optimal Tm) and by titrating MgCl2 concentration (±10% of the standard concentration), using genomic DNA from the attenuated CPV-2 vaccine strain (Cornell, Laboratorios Holland, Jiutepec, Morelos, Mexico) as template. No formal analytical validation (numerical limit of detection, cross-reactivity panel against other parvoviruses or unrelated pathogens) was performed for this in-house assay; specificity is supported indirectly by the 92.9% (13/14) concordance between screening-positive samples and confirmatory VP2-specific PCR/Sanger sequencing (Section 3.2). This finding provides supportive evidence for assay performance but does not constitute formal analytical specificity validation, and this limitation is further acknowledged in the Limitations section. Protoparvovirus-positive samples were subsequently subjected to a CPV-2-specific PCR amplifying a partial (1042-bp) VP2 gene fragment, as previously described [16]. Primer sequences, amplicon sizes, and cycling conditions for all three assays are detailed in Table S3. An attenuated CPV-2 vaccine strain (Cornell, Laboratorios Holland, Mexico) served as the positive control and sterile water as the negative control for both viral PCR assays.

### 2.5. Amplicon Purification and Sanger Sequencing

VP2-positive amplicons were separated on 1% preparative agarose gels, excised, and purified using the Wizard SV Gel and PCR Clean-Up System (Promega, Madison, WI, USA). Bidirectional Sanger sequencing was performed on an Applied Biosystems 3130 Genetic Analyzer (Thermo Fisher Scientific Inc, Waltham, MA, USA) at the DNA Synthesis and Sequencing Unit, Instituto de Biotecnología, Universidad Nacional Autónoma de México (UNAM).

### 2.6. Bioinformatic and Phylogenetic Analysis

Raw chromatogram (.ABI) files were imported into Unipro UGENE v47.0 for Sanger data analysis. VP2 nucleotide sequences (968 bp) were aligned using MUSCLE in MEGA v12.1 [29], trimmed to a common homologous region, and translated into deduced amino acid sequences in AliView v1.30 [30]; the open reading frame was verified, and nucleotide and deduced amino acid sequences were compared against a CPV-2c reference sequence from Uruguay (GenBank accession no. KC196098.1) to identify synonymous and nonsynonymous substitutions, focusing on residues previously associated with CPV antigenic variants (383, 411, 426, 440). Sixty-four representative VP2 reference sequences were retrieved from GenBank. The 30 Mexican sequences comprised all canine CPV-2c VP2 sequences generated and deposited by our research group over the past decade, selected to provide a comprehensive local genetic background against which to compare the novel feline isolates. The remaining 34 sequences (five each from Uruguay, Argentina, Brazil, Peru, Colombia, and Chile; four from the United States, including three coyote-derived sequences) were selected to represent South American and North American CPV-2c diversity, based on prior findings from our group indicating high genetic homology between Mexican and South American strains [15,16,17]; sequences were prioritized by completeness of the VP2 coding region and availability of geographic/host metadata in GenBank. This resulted in a final dataset of 77 aligned VP2 sequences. Pairwise nucleotide distances were calculated in MEGA12 using the *p*-distance model with pairwise deletion.

### 2.7. Recombination Analysis

Potential recombination events within the VP2 alignment were screened using Recombination Detection Program (RDP) version 5.93 [31]. Seven recombination detection methods implemented in the software were applied: RDP, GENECONV, BootScan, MaxChi, Chimaera, SiScan, and 3Seq. Analyses were performed using the default settings, with a highest acceptable *p*-value of 0.05 and Bonferroni correction for multiple comparisons. The analysis included the 77 aligned partial VP2 nucleotide sequences (968 bp) used for phylogenetic reconstruction.

### 2.8. Phylogenetic Analysis

Phylogenetic analysis was performed using the Maximum Likelihood (ML) method implemented in MEGA version 12.1 [29]. The best-fitting nucleotide substitution model was determined using the “Find Best DNA/Protein Models (ML)” procedure implemented in MEGA 12.1, which evaluates multiple candidate substitution models and their variants incorporating rate heterogeneity. Tamura 3-parameter model with invariant sites (T92+I) showed the lowest Bayesian Information Criterion (BIC) value among the models evaluated and was therefore selected for tree reconstruction. Branch support was assessed using 1000 bootstrap replicates. The analysis included 77 aligned VP2 nucleotide sequences (968 bp), and the resulting phylogenetic tree was visualized and edited using FigTree version 1.4.4 [32]. The 13 partial VP2 sequences generated in this study were deposited in GenBank under accession numbers PZ618258 to PZ618270.

### 2.9. Statistical Analysis

Descriptive data were analyzed using GraphPad Prism v10.0.0 (macOS 13.4 Ventura) and Epi Info v7.2.6.0 (Centers for Disease Control and Prevention, Atlanta, GA, USA). Categorical variables were expressed as absolute frequencies and relative frequencies (percentages), and the overall molecular detection rate was calculated as the proportion of protoparvovirus-positive samples relative to the total number of samples tested. For exploratory assessment of potential risk factors, dichotomous exposure variables age category, sex, shared litter box use, cohabitation with other animals, outdoor access, and hunting behaviour were cross-tabulated against CPV-2c PCR status using 2 × 2 contingency tables. Crude odds ratios (ORs) and corresponding 95% confidence intervals (95% CIs) were estimated by standard cross-product ratio calculation; in cases where a zero cell was encountered, a Haldane–Anscombe correction (addition of 0.5 to each cell of the contingency table) was applied to enable OR and 95% CI estimation. Statistical significance of each 2 × 2 comparison was assessed using Fisher’s exact test, two-tailed, given the small expected cell counts in several strata. All statistical tests used a significance threshold set at *p* < 0.05; however, given the exploratory nature of the analysis and the absence of correction for multiple comparisons, results should be interpreted with caution as hypothesis-generating rather than confirmatory.

## 3. Results

### 3.1. Clinical and Demographic Characteristics of the Study Population

**Figure 1:**
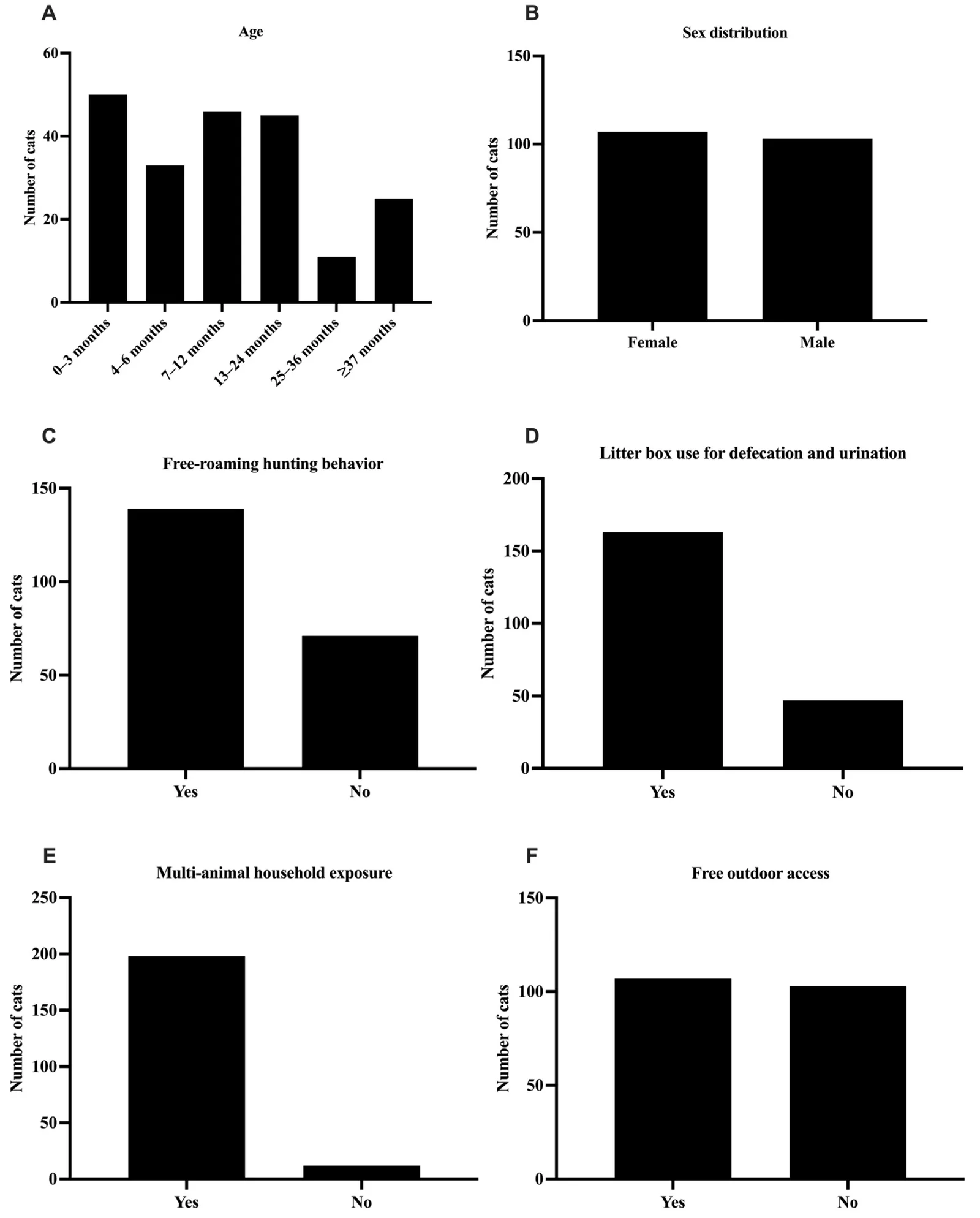
Demographic and management characteristics of the sampled cat population (n = 210). (**A**): age distribution in six categories (0–3, 4–6, 7–12, 13–24, 25–36, and ≥37 months). (**B**): sex distribution (female/male). (**C**): proportion of cats engaging in free-roaming and hunting behavior (Yes/No); this panel depicts the combined free-roaming/hunting exposure question used for descriptive purposes only. In the risk factor analysis, free-roaming/outdoor access and hunting behavior were recorded and analyzed as two independent dichotomous exposures (Table 1). (**D**): indoor litter box use for defecation and urination (Yes/No); for the risk factor analysis, cats using a litter box were further sub-classified according to whether the litter box was shared with at least one other cat (shared, n = 84) or used exclusively/no indoor litter box (not shared, n = 126). (**E**): multi-animal household exposure (Yes/No). (**F**): free outdoor access (Yes/No). Bar charts display absolute counts; CPV-2c-positive animals are indicated in each category.

A general physical examination was performed at enrollment to objectively confirm the ‘apparently healthy’ status of each cat at the time of sampling, rather than relying solely on owner-reported health status. At physical examination, the cohort was predominantly clinically unremarkable, with peripheral lymph nodes, physiological parameters, and mucous membranes within normal limits in 99.0%, 97.6%, and 96.2% of evaluable cats, respectively (Figure S1). The demographic characteristics and management-related exposure variables of the 210 enrolled cats are presented in Figure 1. Regarding age distribution (Figure 1A), cats were distributed across six categories in ascending order: 0–3 months (50/210; 23.8%), 4–6 months (33/210; 15.7%), 7–12 months (46/210; 21.9%), 13–24 months (45/210; 21.4%), 25–36 months (11/210; 5.2%), and ≥37 months (25/210; 11.9%). The 0–3-month group was the most represented, while the 25–36-month stratum was the smallest. Collectively, animals under 12 months of age comprised 61.4% (129/210) of the cohort, reflecting a population enriched with young, potentially immunologically naïve individuals. Sex was nearly equally distributed across the cohort (Figure 1B), with 107 females (51.0%) and 103 males (49.0%), indicating a relatively balanced sex distribution in the study cohort. Regarding free-roaming and hunting behavior (Figure 1C), 66.2% of cats (139/210) were reported to engage in outdoor roaming or hunting activities, whereas the remaining 33.8% (71/210) did not. 77.6% of cats (163/210) used an indoor litter box for defecation and urination (Figure 1D); of these, 84 (51.5%) shared the litter box with one or more conspecifics, while 79 (48.5%) had exclusive access to a private litter box. Multi-animal household exposure (Figure 1E) was highly prevalent, with 94.3% of cats (198/210) living in contact with other animals, and only 5.7% (12/210) kept as single-animal households. Finally, free outdoor access (Figure 1F) was documented in 51.0% of animals (107/210), with the remaining 49.0% (103/210) kept exclusively indoors. The exposure profile depicted in Figure 1 reveals a population characterized by high rates of inter-animal contact, shared litter box use, and free outdoor access.

### 3.2. Protoparvovirus Detection Rate

Protoparvovirus screening by PCR yielded 14 positive samples, corresponding to an overall detection rate of 6.7% (14/210). Of these, 13 samples subsequently amplified a partial VP2 fragment of approximately 1042 bp, consistent with CPV-2, and Sanger sequencing confirmed a nucleotide identity of approximately 99% with CPV-2 reference sequences available in GenBank; the remaining sample could not be further characterized owing to insufficient residual biological material for VP2 amplification and sequencing. Amino acid analysis of the 13 VP2 sequences at the antigenic position 426 revealed the presence of glutamic acid (Glu426; 426E) in all sequences, the defining substitution of the CPV-2c antigenic variant. No FPV-specific sequences were detected among the characterized isolates, indicating that the parvovirus signal molecularly identified in this feline cohort corresponded exclusively to CPV-2c.

### 3.3. Exploratory Risk Factor Analysis

In an exploratory univariate risk factor analysis, shared litter box use, age, vaccination status, free-roaming/outdoor access, and hunting behaviour emerged as informative variables (Table 1). Cats sharing a litter box with at least one other cat (n = 84) showed markedly higher odds of CPV-2c detection (13/84, 15.5%) compared with cats with exclusive or no indoor litter box access (n = 126; 1/126, 0.8%; crude OR 22.89, 95% CI 2.93–178.63, *p* < 0.0001). Younger cats (≤6 months; n = 83) showed a higher detection rate (13.3%, 11/83) than older cats (n = 127; 2.4%, 3/127; crude OR 6.31, 95% CI 1.71–23.39, *p* = 0.0033). Unvaccinated or unknown-vaccination-status cats also showed higher detection (10.1%, 12/119) than vaccinated cats (2.2%, 2/91; crude OR 5.00, 95% CI 1.09–22.94, *p* = 0.0259). Free-roaming/outdoor access and hunting behaviour, analyzed as two independent exposures, were both directly (positively) associated with detection. Cats with free-roaming/outdoor access (n = 107) showed higher detection (10.3%, 11/107) than cats without outdoor access (n = 103; 2.9%, 3/103; crude OR 3.82, 95% CI 1.03–14.11, *p* = 0.0499). Similarly, cats exhibiting hunting behaviour (n = 139) showed higher detection (9.4%, 13/139) than non-hunting cats (n = 71; 1.4%, 1/71; crude OR 7.22, 95% CI 0.93–56.38, *p* = 0.0377). To address potential confounding among the three strongest predictors, a Firth bias-reduced multivariable logistic regression model including shared litter box use, age, and vaccination status was fitted. Shared litter box use remained strongly and independently associated with CPV-2c detection after adjustment (adjusted OR 18.03, 95% CI 3.30–98.58). In contrast, the association between young age and detection was substantially attenuated and no longer statistically significant after adjusting for vaccination status (adjusted OR 3.10, 95% CI 0.79–12.22), consistent with a moderate inverse Pearson correlation between the age category and vaccination status variables in this cohort (r = −0.45, calculated using the same binary coding of both variables as included in the multivariable model). Vaccination status itself showed a protective trend that did not reach statistical significance in the adjusted model (adjusted OR for unvaccinated/unknown vs. vaccinated 3.45, 95% CI 0.72–16.67). Free-roaming/outdoor access and hunting behaviour were not included in the multivariable model given the limited number of events relative to the number of candidate predictors.

### 3.4. Molecular Characterization of the VP2 Gene

Partial VP2 gene sequences (968 bp) were successfully obtained from the 13 CPV-positive feline samples. Translation of the nucleotide sequences resulted in a continuous open reading frame (ORF) without premature stop codons, confirming the integrity of the amplified coding region. For comparative analyses, 64 representative VP2 reference sequences were retrieved from GenBank, resulting in a final dataset of 77 aligned VP2 nucleotide sequences (Supplementary Table S1). Comparison of the deduced amino acid sequences with two historical canine CPV-2c isolates from Mexico (Accession numbers MH705111.1 and MH705103.1) and a representative Uruguayan CPV-2c reference strain (Accession number KC196098.1) revealed limited amino acid variability among the feline isolates (Table 2). Four amino acid residues (383, 411, 426, and 440), previously associated with CPV molecular characterization, were examined. Residue 426 was completely conserved as glutamic acid (E) in all isolates, confirming their classification as the CPV-2c antigenic variant. Likewise, residue 440 remained conserved as alanine (A), consistent with previously reported CPV-2c strains circulating in Mexico and other countries in the Americas [15,16,17,18] (Supplementary Table S1). Variation was detected only at residues 383 and 411. A Q383P substitution was identified in a single feline isolate, whereas an E411G substitution was observed in six of the thirteen isolates. Overall, the amino acid profile of the feline isolates was highly similar to that of contemporary Mexican canine CPV-2c strains, with only minor sequence variation (Table 2). Pairwise nucleotide distance analysis revealed a high degree of sequence conservation among all VP2 sequences included in this study. The 13 feline CPV-2c isolates exhibited pairwise nucleotide identities ranging from 99.38% to 99.90% (mean, 99.66%), indicating high genetic similarity among the feline CPV-2c sequences obtained in this study. Similarly, comparisons with canine-derived CPV-2c reference sequences from Mexico and other American countries showed consistently high sequence identities, further supporting the close genetic relationship among CPV-2c strains circulating throughout the Americas. In contrast, the Colombian reference sequences, classified as CPV-2a, exhibited lower nucleotide identities with the feline isolates, indicating that the reduced similarity reflects differences between viral variants rather than geographic origin.

### 3.5. Recombination Analysis

No evidence of recombination was detected within the 968-bp VP2 alignment. None of the seven methods implemented in RDP5 (RDP, GENECONV, BootScan, MaxChi, Chimaera, SiScan, and 3Seq) found recombination events among the 77 sequences analyzed. Therefore, no sequences were excluded from the subsequent phylogenetic analysis.

### 3.6. Phylogenetic Analysis

**Figure 2:**
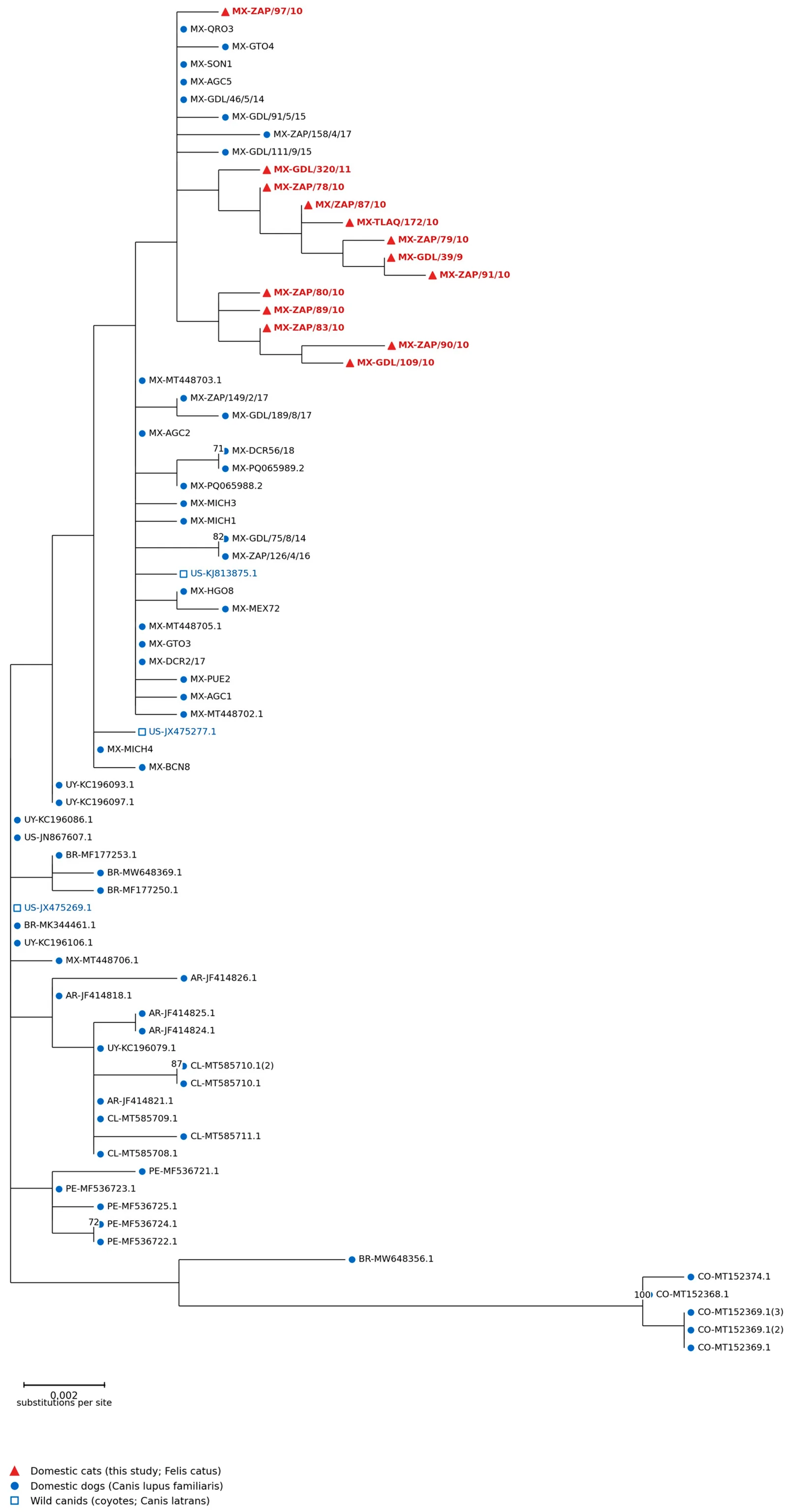
Phylogenetic analysis of partial VP2 gene sequences of Carnivore protoparvovirus 1. The phylogenetic tree was inferred using the Maximum Likelihood method based on partial VP2 nucleotide sequences. Feline CPV-2c sequences generated in the present study are indicated by red triangles. GenBank reference sequences were obtained from canids and consisted predominantly of domestic dog isolates (*Canis lupus familiaris*); three sequences derived from coyotes (*Canis latrans*) are indicated by open blue squares. Domestic dog reference sequences are indicated by blue circles. Bootstrap values ≥ 70% are shown at the corresponding nodes. The scale bar represents the number of nucleotide substitutions per site.

A Maximum Likelihood phylogenetic tree was reconstructed using the 13 feline VP2 sequences generated in this study together with 64 reference VP2 sequences retrieved from GenBank, comprising 30 sequences from Mexico, five each from Uruguay, Argentina, Brazil, Peru, Colombia, and Chile, and four from the United States, including three coyote-derived sequences (Figure 2). The sequences generated in this study clustered most closely with previously reported Mexican canine CPV-2c isolates and were also closely related to CPV-2c strains from the United States, Uruguay, Argentina, Brazil, Chile, and Peru. The three coyote-derived sequences included in the analysis were distributed within the same CPV-2c clade and did not constitute an independent evolutionary lineage. Although several internal nodes were supported by moderate to high bootstrap values, no consistent phylogenetic clustering according to host species was observed. Overall, the phylogenetic reconstruction indicates that the feline isolates belong to the same genetically conserved CPV-2c population circulating among domestic dogs, wild canids, and domestic cats throughout the Americas, providing no evidence of a feline-specific evolutionary lineage.

## 4. Discussion

The present study constitutes the first molecular investigation of Carnivore protoparvovirus 1 in clinically healthy domestic cats in Mexico and, to our knowledge, in Latin America. The overall molecular detection rate of 6.7% (14/210) in blood samples, with the exclusive identification of CPV-2c (Glu426), provides evidence of CPV-2c DNA detection in apparently healthy cats in the Guadalajara Metropolitan Area (GMA). It should be emphasized that blood positivity indicates viral DNA presence, consistent with viremia or leukocyte-associated viral persistence, and does not, by itself, demonstrate infectiousness or transmission capacity; this distinction is relevant when interpreting the epidemiological significance of these findings. The 6.7% molecular detection rate falls within the lower range reported in asymptomatic cats elsewhere. Balboni et al. (2018) detected protoparvovirus DNA in 16.7% of buffy coat samples from healthy cats in Sardinia, Italy [26], while Clegg et al. (2012) reported fecal shedding prevalences of ~33% in UK shelter cats [20], and Rehme et al. (2022) documented 48.7% in German shelters [21]. Byrne et al. (2018) found no CPV shedding in Australian shelter cats [33]. This variability likely reflects differences in sample type (blood vs. feces), diagnostic sensitivity, shelter versus community settings, and local viral epidemiology. Moreover, a recent study in apparently healthy dogs from the Campania region, Italy, reported a comparable molecular detection rate of 6.5% (11/117) for Carnivore protoparvovirus 1 (CPV-2) in fecal samples, alongside detection of other, more recently described canine parvoviruses [34], supporting the notion that subclinical viral DNA carriage is not unique to cats but represents a broader phenomenon among apparently healthy companion carnivores. Several non-mutually exclusive explanations could account for the positive PCR results observed in the present feline cohort: (i) early-phase viremia preceding seroconversion and viral clearance; (ii) passive elimination of residual viral DNA following prior infection and immune resolution, without ongoing active replication; or (iii) transient detection of vaccine-derived viral DNA. The latter possibility is considered unlikely in this study, as variant classification relied on VP2-specific PCR and Sanger sequencing rather than antigen detection alone, and all 13 sequenced isolates were unambiguously classified as wild-type CPV-2c (Glu426) rather than the vaccine strain used as a positive control (Cornell strain, CPV-2b origin). Nonetheless, distinguishing between these scenarios would require longitudinal sampling and viral load quantification, which were beyond the scope of this cross-sectional design. Notably, our use of whole blood may detect viremia or leukocyte-associated viral persistence rather than active intestinal shedding, potentially capturing a different infection phase and yielding a more conservative estimate [26].

The absence of FPV and the exclusive identification of CPV-2c mirror the canine epidemiological landscape in the GMA, where CPV-2c has been the sole circulating variant since at least 2014 [15,16,17]. Our findings suggest that the high CPV-2c circulation documented in the local canine population may contribute to CPV-2c circulation in the feline population in this setting, consistent with reports of emerging CPV-2c feline infections in Egypt [22,23] and India, regions where CPV-2c also dominates in dogs.

Shared litter box use emerged as the most consistent management-related risk factor, with cats using shared litter boxes showing markedly higher odds of CPV-2c detection than those defecating outdoors or in other locations, underscoring the central role of fecal–oral and fomite-mediated transmission in parvoviral spread [6,21]. Free-roaming/outdoor access and hunting behavior were also positively associated with CPV-2c detection, reinforcing the central role of environmental and fecal–oral exposure routes already suggested by the shared litter box findings. Cats with outdoor access or hunting behavior likely encounter a wider range of contaminated environments, including feces from other cats, dogs, or wild carnivores in shared outdoor spaces, which may increase the probability of viral DNA exposure beyond that afforded by indoor shared litter box use alone. This interpretation is biologically consistent with the recognized environmental persistence and fecal–oral transmission route of protoparvoviruses and complements rather than contradicts the litter-box-associated risk described above. Nevertheless, given the modest number of events and wide confidence intervals (particularly for hunting behavior, whose 95% CI includes 1), these associations should still be interpreted as hypothesis-generating rather than confirmatory. Young age (≤6 months) showed a significant crude association with CPV-2c detection (OR 6.31; *p* = 0.0033), consistent with the recognized immunological naivety of juvenile cats [19]. However, as noted above, this association was attenuated and lost statistical significance after adjustment for vaccination status in the multivariable Firth model (adjusted OR 3.10; 95% CI 0.79–12.22), suggesting that incomplete vaccination coverage, rather than age per se, may be the more proximal risk factor underlying the crude association. Overall, these risk factor findings should be interpreted as hypothesis-generating, given the cross-sectional design, modest sample size, and wide confidence intervals. While the present study already incorporates Firth-adjusted multivariable analysis for the three strongest predictors (shared litter box use, age, and vaccination status), confirmation in larger longitudinal studies incorporating additional covariates such as household composition and dog cohabitation remains warranted.

This study demonstrates that apparently healthy domestic cats from the GMA harbor CPV-2c viruses that are genetically similar to those circulating in domestic and wild canids throughout the Americas. The conservation of the diagnostic residue Glu-426 confirmed that all feline viruses belonged to the CPV-2c antigenic variant, whereas residue 440 also remained fully conserved. Only two amino acid substitutions (Q383P and E411G) were detected within the analyzed VP2 fragment, but these changes did not affect the residues currently used for CPV variant classification. These findings indicate that the feline isolates exhibit the same molecular characteristics previously described for contemporary canine CPV-2c strains [15,16,17] the available data are insufficient to determine whether these amino acid changes are associated with host-specific adaptation, and they most likely represent naturally occurring genetic variation. Similar shared lineages between dogs and cats have been described in the UK [20] and Italy [26]. The phylogenetic reconstruction and pairwise nucleotide identity analysis consistently supported this conclusion. The feline isolates were inserted among canine-derived CPV-2c strains from Mexico and other American countries rather than forming a separate host-associated lineage. Likewise, the high nucleotide identity observed among the feline isolates (99.38–99.90%; mean 99.66%) and between feline and canine CPV-2c sequences indicates that these viruses belong to a genetically conserved viral population. The lower nucleotide identity observed for the Colombian reference sequences is explained by their classification as CPV-2a rather than CPV-2c, indicating that this difference reflects antigenic variant divergence rather than geographic separation. The absence of host-specific phylogenetic clustering, together with the close genetic relationship among feline, canine, and coyote-derived viruses, supports the hypothesis that CPV-2c circulates among susceptible carnivore hosts without clear host-associated genetic segregation. Importantly, no evidence of recombination was detected within the 968-bp VP2 region analyzed, indicating that no detectable recombination signal was present in the sequence dataset used for phylogenetic reconstruction.

## 5. Conclusions

To our knowledge, this is the first molecular study reporting CPV-2c detection in apparently healthy domestic cats in Mexico and contributes to the limited molecular evidence available from Latin America. CPV-2c DNA was detected in 6.7% of sampled cats; the sequenced isolates showed high genetic similarity to local canine strains and were phylogenetically related to South American lineages, indicating a shared, cross-species viral population without evidence of feline-specific adaptation or recombination. Shared litter box use, free-roaming/outdoor access, and hunting behavior were independently associated with increased odds of detection, pointing to fecal–oral and environmental exposure as the principal transmission routes, while the crude association with young age appeared to be driven mainly by incomplete vaccination coverage rather than age itself. Because viral DNA was detected only in blood, these findings demonstrate the presence of CPV-2c DNA in blood from apparently healthy cats, but do not establish active infection, viral shedding, or infectiousness. Nonetheless, they underscore the need to incorporate cats into CPV-2 molecular surveillance programs that have traditionally focused exclusively on dogs, particularly in regions where CPV-2c is endemic in the canine population. Future longitudinal studies incorporating fecal sampling, viral load quantification, and assessment of dog-cat cohabitation are warranted to determine whether cats contribute actively to CPV-2c transmission and to evaluate the cross-protective efficacy of current feline panleukopenia vaccines against this canine-origin variant.

## 6. Limitations of the Study

The cross-sectional design cannot determine viremia duration or transmission direction. Whole blood, rather than feces, limits inferences about intestinal shedding and infectiousness; conventional PCR precluded viral load estimation, and incomplete vaccination records precluded assessment of vaccine-derived interference. Recombination screening covered only the 968-bp VP2 fragment; events elsewhere in the genome cannot be excluded, and whole-genome analysis would be needed to fully assess recombination’s evolutionary role. Nonetheless, the consistent amplification, sequencing, and phylogenetic placement of the 13 VP2 sequences within the CPV-2c clade confirm CPV-2c as the variant identified among the 13 successfully characterized samples. Because fecal shedding, environmental contamination, and direct cat–dog transmission were not assessed, this study cannot establish onward transmission capability; this remains open for studies combining fecal sampling and viral load quantification. Detection of subclinical CPV-2c DNA nonetheless supports including cats in CPV-2 surveillance programs traditionally focused on dogs [19]. Future longitudinal studies should quantify fecal shedding duration and viral loads by qPCR, and evaluate cross-protective efficacy of current FPV-based vaccines in cats. Selection bias is a further concern. Excluded and included samples could not be formally compared demographically, as questionnaire data were incomplete for excluded animals. The convenience multi-site strategy also enriched the cohort for young animals (61.4%, 129/210, under 12 months), reflecting recruitment at spay/neuter units and a feral cat rescue center; neither the detection rate (6.7%) nor the age-related odds ratio should be extrapolated to the broader municipal population without caution. The in-house screening assay was designed via in silico primer verification (NCBI Primer-BLAST) and empirically optimized for annealing temperature and MgCl2 concentration, but lacked formal analytical validation: limit of detection, a cross-reactivity panel, and inter-assay reproducibility were not determined, a common limitation of newly designed in-house assays. Finally, because the screening PCR (FTA1/FTA2) cannot itself distinguish FPV from CPV-2, variant assignment relied on a second, CPV-2-specific PCR and sequencing of VP2 residue 426. Without a direct FPV-specific confirmatory assay, the single screening-positive sample that failed CPV-2-specific amplification cannot be formally excluded as FPV or a divergent template, rather than reflecting insufficient material as assumed. Future studies should include parallel FPV-specific PCR for direct variant differentiation.

## Figures and Tables

**Table 1:** Exploratory univariate and Firth bias-reduced multivariable analysis of management and demographic factors associated with Carnivore protoparvovirus 1 (CPV-2c) detection in apparently healthy domestic cats (n = 210) from the Guadalajara Metropolitan Area, Mexico.

Variable	Category (Exposed vs. Unexposed)	Exposed CPV-2c+/n (%)	Unexposed CPV-2c+/n (%)	Crude OR (95% CI) ^1^	Firth-Adjusted OR (95% CI) ^2^
Shared litter box use	Shared vs. not shared/no litter box ^3^	13/84 (15.5)	1/126 (0.8)	22.89 (2.93–178.63) ****	18.03 (3.30–98.58)
Age	≤6 months vs. >6 months	11/83 (13.3)	3/127 (2.4)	6.31 (1.71–23.39) **	3.10 (0.79–12.22) ^4^
Vaccination status	Unvaccinated/unknown vs. vaccinated	12/119 (10.1)	2/91 (2.2)	5.00 (1.09–22.94) *	3.45 (0.72–16.67) ^5^
Free-roaming/outdoor access	Yes vs. No	11/107 (10.3)	3/103 (2.9)	3.82 (1.03–14.11) *	Not modeled ^6^
Hunting behavior	Yes vs. No	13/139 (9.4)	1/71 (1.4)	7.22 (0.93–56.38)	Not modeled ^6^
Dog cohabitation	Yes vs. No	ND	ND	Not available	Not available ^7^

^1^ Crude odds ratios (ORs) and 95% confidence intervals (CIs) estimated by cross-product ratio; Haldane–Anscombe correction (+0.5/cell) applied where a zero cell was present; two-tailed Fisher’s exact test, * *p* < 0.05, ** *p* < 0.01, **** *p* < 0.0001; results are exploratory, without correction for multiple comparisons. ^2^ Firth bias-reduced (penalized-likelihood) multivariable logistic regression including shared litter box use, age (≤6 months), and vaccination status simultaneously (n = 210, 14 events); used given the small number of events and quasi-complete separation observed in the crude tables. ^3^ Unexposed group comprises cats with exclusive litter box access plus cats without an indoor litter box. ^4^ After adjustment for vaccination status, the association between young age and CPV-2c detection was attenuated and no longer statistically significant, consistent with a moderate inverse correlation between age and vaccination coverage (r = −0.45) in this cohort. ^5^ Adjusted OR expressed as unvaccinated/unknown vs. vaccinated for consistency of direction with the crude estimate; 95% CI includes 1. ^6^ Not included in the multivariable model given the limited number of events relative to the number of candidate predictors. ^7^ Dog cohabitation was not recorded in the original questionnaire for any of the 210 cats and is declared as a data limitation. ND: not determined.

**Table 2:** Comparison of deduced VP2 amino acid residues among feline isolates and representative reference strains.

Sequence	GenBank Accession	383	411	426	440
Uruguay CPV-2c reference	KC196098.1	Q	E	E	T
Historical Mexican strain	MH705111.1	Q	E	E	A
Historical Mexican strain	MH705103.1	Q	E	E	A
MX-GDL/39/9	PZ618258	Q	G	E	A
MX-ZAP/78/10	PZ618259	Q	E	E	A
MX-ZAP/79/10	PZ618260	Q	G	E	A
MX-ZAP/80/10	PZ618261	Q	E	E	A
MX-ZAP/83/10	PZ618262	Q	E	E	A
MX-ZAP/87/10	PZ618263	Q	G	E	A
MX-ZAP/89/10	PZ618266	Q	E	E	A
MX-ZAP/90/10	PZ618267	Q	G	E	A
MX-ZAP/91/10	PZ618264	Q	E	E	A
MX-ZAP/97/10	PZ618265	Q	G	E	A
MX-GDL/109/10	PZ618268	Q	E	E	A
MX-TLAQ/172/10	PZ618270	P	G	E	A
MX-GDL/320/11	PZ618269	Q	E	E	A

Residue 426 (E) *identifies the CPV-2c antigenic variant. E411G was detected in six feline isolates, whereas Q383P was detected in one isolate*.

## Data Availability

The original contributions presented in this study are included in the article/supplementary material. Further inquiries can be directed to the corresponding author.

## Supplementary Materials

The following supporting information can be downloaded at: https://www.mdpi.com/article/10.3390/vetsci13090977/s1, Figure S1: Clinical status of enrolled cats at physical examination; Table S1: VP2 sequences included in the molecular and phylogenetic analyses; Table S2: Comparison of deduced VP2 amino acid residues among feline CPV-2c isolates and reference strains; Table S3: Consolidated PCR assay conditions.
