# Supplementary material for: Molecular Detection and Phylogenetic Characterization of CPV-2c in Apparently Healthy Domestic Cats in the Guadalajara Metropolitan Area, Mexico
Source: Vet Sci. 2026 Sep 17;13(9):977. doi: 10.3390/vetsci13090977 (PMC13612087; doi:10.3390/vetsci13090977)

Figure S1. Clinical status of enrolled cats at physical examination. Bar charts showing the number of cats with peripheral lymph nodes (A), physiological parameters (B), and mucous membranes (C) classified as within normal limits or altered at the time of blood sampling (n = 210; physiological parameters n = 167 due to missing records).

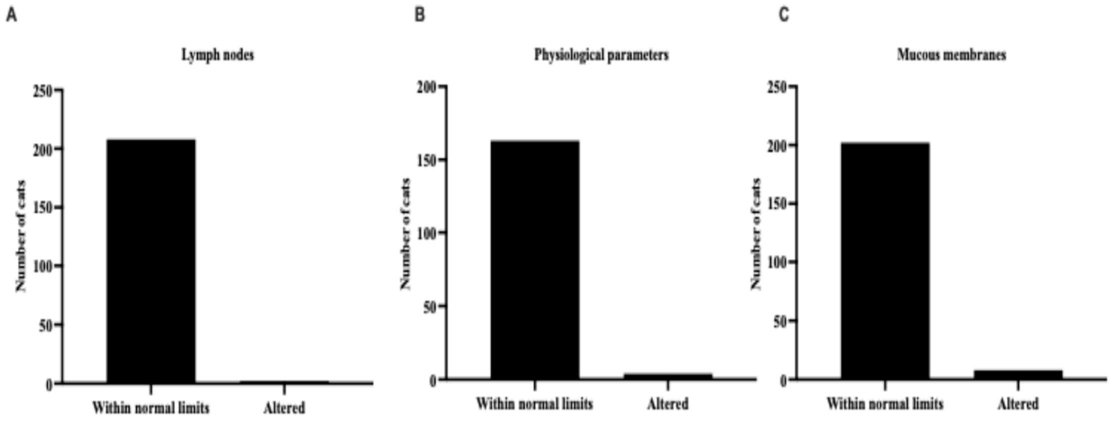

Supplement: Supplementary file 1 [file vetsci-13-00977-s001.zip › Figure S1.pdf]
