# Supplementary material for: Molecular Detection and Phylogenetic Characterization of CPV-2c in Apparently Healthy Domestic Cats in the Guadalajara Metropolitan Area, Mexico
Source: Vet Sci. 2026 Sep 17;13(9):977. doi: 10.3390/vetsci13090977 (PMC13612087; doi:10.3390/vetsci13090977)
Supplement: Supplementary file 1 [file vetsci-13-00977-s001.zip › Table_S3_Consolidated_PCR_Conditions.pdf]

Table S3. Consolidated PCR assay conditions used in this study.

Assay (target)	Primers (5'->3')	Amplicon (bp)	Cycling conditions	Reference
β-globin DNA quality control	B1-F: ACCACCAACTTCATCCACGTTCACC B2-R: CTTCTGACACAACTGTGTTCACTAGC	140	95°C 10min; 35x(94°C 1min, 52°C 30s, 72°C 1min); 72°C 7min	[24,25]
Protoparvovirus screening (FPV/CPV-2)	FTA1: AGAGCATTGGGCTTACCACC FTA2: CCCATTTGAGTTACACCACGTC	150	94°C 5min; 25x(94°C 30s, 59°C 30s, 72°C 30s); 72°C 7min	[PENDING - see Response to Comment 2]
CPV-2 specific VP2 amplification	VP2-F: GGAAACCAACCATACCAACTCC VP2-R: GGATTCCAAGTATGAGAGGC	1042	94°C 5min; 35x(94°C 30s, 50°C 1min, 72°C 1min); 72°C 10min	[12,13]

Master mix components (buffer, dNTPs, MgCl2, Taq polymerase, template volume) were assay-specific and are retained in the main-text Methods narrative only where they diverge from standard conditions.
